# A Case of Synchronous Squamous Cell Carcinoma in the Esophagus and Stomach: A Rare Duo

**DOI:** 10.14740/gr628w

**Published:** 2015-02-14

**Authors:** Pravir Gambhire, Vinay Zanvar, Ashok Mohite, Sunil Pawar, Aniruddha Chafekar, Pravin Rathi

**Affiliations:** a Department of Gastroenterology, Topiwala National Medical College & B.Y.L Nair Hospital, Mumbai 400008, India; b Department of Surgery, Topiwala National Medical College & B.Y.L Nair Hospital, Mumbai 400008, India

**Keywords:** Endoscopy, Chemotherapy, Esophageal carcinoma, Stomach carcinoma, Synchronous, GIT

## Abstract

Synchronous squamous cell esophageal and squamous cell gastric cancer is a rare duo. A 48-year-old male visited our hospital with a history of dysphagia and melena and was diagnosed with synchronous esophageal and gastric cancer by endoscopy and histopathology. We report a case of a synchronous cancer that was successfully treated by chemotherapy followed by surgery. We also discuss the hypothesis regarding the origin and presentation of the synchronous cancer in the GIT.

## Introduction

A synchronous cancer of squamous cell origin found in the esophagus and stomach is rare among gastrointestinal cancers. In Japan, esophageal squamous cell carcinoma is frequently associated with adenocarcinoma of the stomach [1]. Squamous cell carcinomas take up 38% of all esophageal cancers, and it is common in esophagus, but not in stomach [2], less than 100 cases were reported until now [3]. We had a case of squamous cell carcinoma of esophagus and stomach and therein we report this case. Although the optimal management of simultaneous gastric and esophageal cancer is not established yet, principle of treatment is radical resection of each cancer, such as total gastrectomy with esophagectomy [4]. We report here a case of a patient with synchronous esophageal and gastric cancer who achieved and sustained complete remission following combination chemotherapy and was operated thereafter.

## Case Report

A 48-year-old male patient admitted with complaints of difficulty in swallowing, easy fatigability and generalized weakness for 4 months, along with melena intermittently. He also had anorexia and a weight loss of 14 kg. His social history was significant for drinking about 40 g of alcohol daily and smoking one pack of cigarettes for 20 years. He had history of blood transfusion in recent past for anemia. His brother died due to advanced stomach cancer.

**Figure 1:**
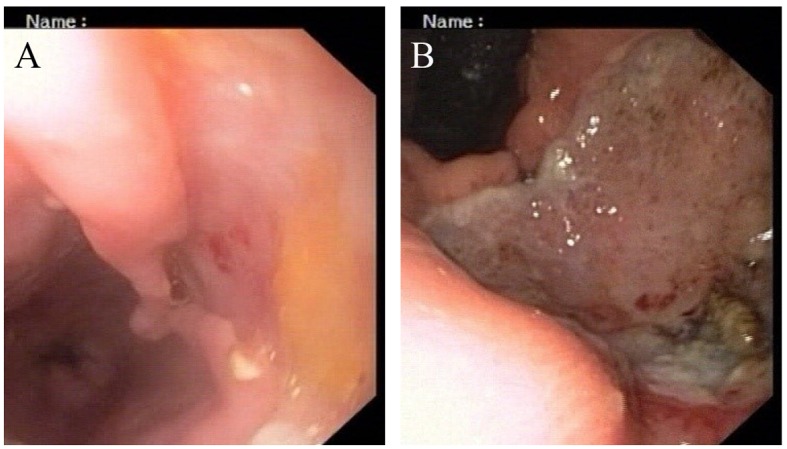
(A, B) Endoscopic appearance of esophageal and gastric lesion.

**Figure 2:**
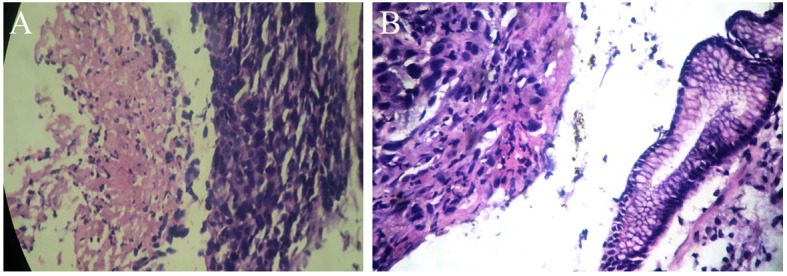
(A, B) Histopathologic examination of esophageal and stomach mass after H&E staining under × 40 magnification respectively showing squamous cell carcinoma.

**Figure 3:**
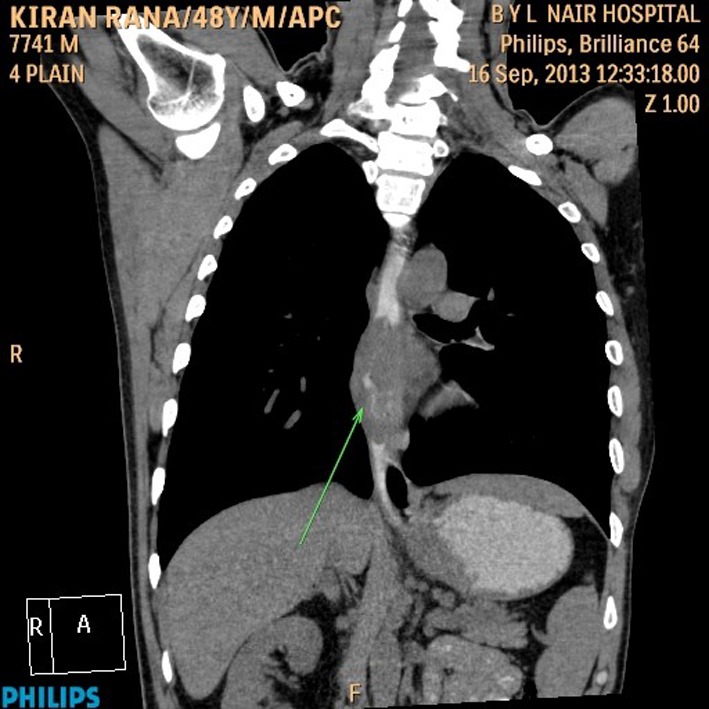
CT scan of abdomen and thorax.

His general examination revealed pallor and pulse of 120/min. Other systemic examination was within normal limit. The complete blood count showed white blood cell count 9.6 × 10^9^/L, Hb 64 g/L, MCV 65 fL, MCH 21 pg/cell, and MCHC 308 g/L. Platelet count was 284 × 10^9^/L. His blood chemistry was normal. Stool occult blood was positive. The chest X-ray and USG abdomen showed no abnormality. The endoscopic finding revealed an irregular ulceroinfiltrative lesion involving half of circumference in mid esophagus with lesion extending from 28 to 35 cm from incisors (Fig. 1A) and another ulcerated mass along the lesser curvature in the body of stomach (Fig. 1B). Histopathology examination showed moderately differentiated squamous cell carcinoma of esophagus (Fig. 2A) with poorly differentiated squamous cell carcinoma of stomach (Fig. 2B). CT scan of abdomen and thorax showed concentric mass involving subcarinal esophagus for a length of 7 cm with loss of fat planes between tumor and aorta. Another mass was noted in body of stomach along lesser curvature with loss fat planes between pancreas (Fig. 3).

**Figure 4:**
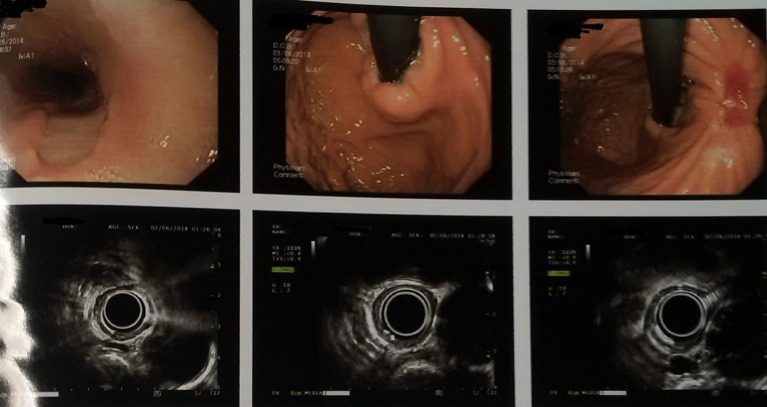
Endoscopic ultrasonography images (post chemotherapy).

The patient was given palliative chemotherapy with docetaxel and cisplatin. He received three cycles of chemotherapy, without any severe toxic side effects. After three cycles of chemotherapy, the endoscopic examination showed significant disappearance of both the esophageal mass and the stomach cancer, and only a remnant clean base ulcer in esophagus and ulcer at incisura (Fig. 4). Endosonography was suggestive of loss of layer pattern with thickening in mid part of esophagus, which appeared adherent to pleura, with right subcarinal node and was free from aorta. These follow-up images revealed a good response to chemotherapy. At that point of time, surgical resection was planned. Tri-incisional esophagectomy was undertaken. A right posterolateral thoracotomy was performed and *en bloc* resection was performed. Stomach was mobilized through laparotomy for the construction of the gastric conduit. Esophagogastric anastomosis was then performed via left neck incision. Postoperative pathologic findings demonstrated complete histological disappearance of both the lesions.

This combination chemotherapy was continued further for three cycles, with close follow-up examinations.

## Discussion

There have been a few reports on the double primary cancers consisting of esophageal squamous cell carcinomas and gastric adenocarcinomas, but squamous cell carcinoma of stomach is a rare finding as described in this report [5] recently. The prevalence of squamous cell carcinoma is on the decline, but still common in Asia in association with smoking and alcohol consumption [6]. However, it is not easy to explain the simultaneous occurrence of two malignancies in different but adjacent parts of the upper digestive tract, considering that the esophagus and the stomach have different histological structures and the epidemiology is different. Generally, the synchronous occurrence of cancers has been explained by the concept of field carcinogenesis. The esophageal and gastric cancers share some risk factors, including diet, low socioeconomic status, age, alcohol and tobacco use and nitrate exposure [6]. Our patient was indeed a heavy alcohol consumer.

To diagnose a synchronous cancer, three conditions should be satisfied. First of all, cancer lesions should be located apart from each other, and should not be contiguous. Secondly, they must be pathologically different. Thirdly, both lesions should have mucosal lesions. That is, the cancer originates from two lesions: one from the esophagus and the other from the gastric body. Moreover, the fact that the level of differentiation was different in two lesions suggests that they arise from different origins. Valduga et al [7] reported a synchronous cancer with undifferentiated squamous cell carcinoma of stomach and well-differentiated squamous cell carcinoma of esophagus. In this regard, the main lesion of the synchronous cancer occurred from the fundus of the stomach reaching the esophagogastric junction, and the lower esophagus. Squamous cell carcinoma mainly occurs in the mid to lower esophagus, and it is not uncommon to have other accompanying cancerous lesions. Suzuki et al [8] reported that the most common lesion is the stomach (59.6%), and then colon and rectum (12.3%) in the order of frequency. Therefore, it is important to consider the possibility of other existing cancers other than the esophagus.

Though optimal management of the simultaneous gastric and esophageal cancers has not been established yet, radical resection for both cancers, i.e. esophagectomy with total gastrectomy was usually recommended [4]. II study of docetaxel plus cisplatin as first-line therapy in patients with metastatic squamous esophageal cancer showed the response rate of 33.3%, the median progression free survival of 5.0 months and the median overall survival rate of 8.3 months [9]. In addition, in metastatic or locally advanced gastric adenocarcinoma, this combination chemotherapy showed very potent efficacy [10]. Chemotherapy plays a major role in the palliative therapy and is still the primary mode of treatment for the recurrent metastatic esophageal or gastric cancer. The presented case was a rare report synchronous cancer of squamous cell origin in the esophagus and stomach with successful therapy using surgery post neoadjuvant chemotherapy.
